# Surveillance Imaging in Cutaneous Squamous Cell Carcinoma: A Retrospective Analysis Informing Surveillance Recommendations

**DOI:** 10.21203/rs.3.rs-9205484/v1

**Published:** 2026-05-14

**Authors:** Monika Bapna, Ann W. Silk, Emily Karn, Justine V. Cohen, Karam Khaddour, Jeffrey P. Guenette, Emily S. Ruiz

**Affiliations:** Brigham and Women’s Hospital; Dana-Farber Cancer Institute; Brigham and Women’s Hospital; Dana-Farber Cancer Institute; Dana-Farber Cancer Institute; Brigham and Women’s Hospital; Harvard Medical School

**Keywords:** cutaneous squamous cell carcinoma (cSCC), imaging for cSCC, high stage cSCC recurrence, surveillance imaging protocols, metastatic cSCC

## Abstract

**Background:**

Imaging surveillance for high-stage (HS), regionally metastatic (RM), and unresectable (UR) cutaneous squamous cell carcinoma (CSCC) is not standardized, and evidence guiding post-treatment imaging is limited.

**Objective:**

To characterize imaging surveillance practices for HS, RM, and UR CSCC at a single high-volume cancer center and propose tailored surveillance protocols.

**Methods:**

Retrospective cohort study of patients with HS, RM, or UR CSCC discussed at a multidisciplinary tumor board (January 2021-November 2024).

**Results:**

For HS CSCC, CT of the regional nodal basin was most common (83.9%), typically every 6 months for 2 years (61.3%). RM CSCC most frequently underwent CT (64.7%) or PET/CT (35.3%), most commonly every 6 months for 3 years (67.6%). UR CSCC surveillance included CT (57.9%) and PET/CT (42.1%), most often every 3 months for year 1 and every 6 months for years 2–3 (52.6%).

**Conclusions and Relevance:**

Imaging surveillance practices for advanced CSCC varied by disease extent and treatment. Proposed surveillance recommendations include CT every 6 months for 2 years (HS), CT every 6 months for 3 years (RM), and CT or PET/CT every 3 months for year 1 then every 6 months in years 2–3 (UR). These findings reflect real-world practice and may inform development of structured surveillance protocols.

## Introduction

Cutaneous squamous cell carcinoma (CSCC) represents the second most prevalent skin cancer in the United States, and is often cured with surgery alone. The National Comprehensive Cancer Network (NCCN) guidelines recommend clinical monitoring of patients with CSCC every 3-12 months for 2 years, then every 6-12 months for 3 years, and annually for life. For surveillance, the NCCN guidelines recommend imaging of regional nodal basin and to evaluate for distant metastatic disease, ideally based on multidisciplinary board recommendation, or as clinically indicated.^1^ While most cases follow a favorable course, a subset of tumors will develop local invasion, metastasis or lead to disease-related death. However, data on optimal imaging surveillance protocols for advanced CSCC remain limited.

Several retrospective studies have shown imaging to be valuable for high stage CSCCs. Diagnostic imaging at the time of initial treatment can aid in evaluating local tumor extension and metastasis. In a retrospective analysis of 98 patients with 108 high-stage CSCCs, 48% received imaging as part of their evaluation. Among those, 50% underwent imaging on or before the day of primary treatment, while 27% had imaging performed within 30 days of treatment. Notably, imaging influenced primary and/or adjuvant management decisions in 33% of cases^2^. Interestingly, imaging was associated with a 50% reduction in the risk of nodal metastasis (NM) and any disease-related outcomes (DRO), which was attributed to the early detection and management of advanced disease.

Surveillance imaging has been shown to be helpful for identifying recurrences in select CSCCs. One study of 87 tumors that underwent baseline and/or surveillance imaging found that imaging identified subclinical disease in 21% of high-risk CSCC cases and more than half of the 18% of NMs were detected on surveillance imaging within the first two years post-treatment^3^. The German Dermatological Society’s updated guidelines on CSCC provide general timeframes for surveillance imaging based on risk stratification^4^. However, these recommendations are largely based on expert opinion and underscore a broader gap in evidence-based guidance, particularly as no standardized surveillance imaging protocols exist for CSCC in the United States.

The goal of this study is to evaluate imaging surveillance practices for patients with high stage (HS), fadjuvant regionally metastatic (RM) and unresectable (UR) CSCC at a single high volume cancer center and to utilize this data to propose tailored protocols for advanced CSCC.

## Methods

This study was approved by the Mass General Brigham institutional review board. Patients were eligible for inclusion if they had been reviewed by a multidisciplinary tumor board comprised of dermatologists, medical oncologists, radiation oncologists, head and neck surgeons, and radiologists. All patients who completed treatment for a HS/RM/UR-CSCC from January 2021 through November 2024 and had documentation of surveillance recommendations were included in the analysis. HS tumors were defined as BWH stage T2b/T3 CSCC or those with BWH stage T2a (i.e., only one high-risk feature) with other known risk features such as local recurrence or incompletely excised tumors^5^. RM included metastasis in the draining nodal basin (NM), intraparotid metastasis (IPM) or in-transit metastasis (ITM). UR-CSCC was defined as disease deemed inoperable by a multidisciplinary team. Patients currently on treatment or with no documentation of surveillance plans were excluded.

Patient demographics, tumor characteristics, treatments, imaging modalities (computed tomography (CT), positron emission tomography (PET)/CT, magnetic resonance imaging (MRI), and ultrasonography (US)), and surveillance protocols were extracted from electronic medical records. Surveillance protocols were analyzed to assess imaging frequency, modality, and duration stratified by disease level (i.e. high stage, locally advanced and metastatic) and treatment. Descriptive statistics were used to summarize patient and tumor characteristics.

Based on the data, proposed surveillance imaging protocols were developed that included imaging modality, frequency, and duration. In addition, a list of clinical scenarios that would lead to deviations from the proposed surveillance imaging protocols was generated based on the data extracted from the retrospective cohort.

## Results

A total of 84 patients, predominantly male (n=68, 81%), with a mean age of 74.4 years (±10.1 years) were included (Table I). 25 (29.8%) patients were immunosuppressed (hematologic malignancy: n=15, 60%; solid organ transplant: n=8, 32%; rheumatoid arthritis: n=1, 4%, chronic prednisone use: n=1, 4%). Of the 84 patients, 31 (36.9%) were HS, 34 (40.5%) were RM, and 19 (22.6%) were UR.

### High Stage Disease

Among the 31 patients with HS tumors, BWH staging classified 5 (16.1%) as T2a, 22 (71%) at T2b, and 4 (12.9%) at T3 cases. The 5 T2a tumors were included in the analysis for the following reasons: locally recurrent tumor (n=3, 60%), positive excision margins in the original tumor with no additional surgery or adjuvant treatment (n=1, 20%), and large caliber PNI (n=1, 20%). According to AJCC 8 staging 8^th^ edition, there were 2 (6.5%) recurrent T1 tumors, one (50%) with small caliber perineural invasion (PNI); 8 (25.8%) T2 of which 4 (50%) were greater than 2cm and 4 (50%) were greater than 2cm and had small caliber PNI; 20 (64.5%) T3; and 1 (3.2%) T4a.

For treatment, 10 (32.3%) underwent surgery monotherapy, of which 7 (70%) tumors would typically be considered for adjuvant radiation therapy (ART), but 4 (40%) deferred ART due to personal preference and 3 (30%) had a recurrence in a radiated field so could not get additional radiation. 21 (67.7%) tumors underwent surgery and either adjuvant or neoadjuvant therapy: 17 (81%) received ART, 2 (9.5%) received neoadjuvant cemiplimab, 1 (4.8%) received neoadjuvant cemiplimab and ART, and 1 (4.8%) received neoadjuvant and adjuvant cemiplimab.

The most common surveillance imaging modality for HS tumors was CT of the regional nodal basin regardless of treatment (n=26, 83.9%) (Table II). Of these 26 patients, 8 (30.8%) were also enrolled in a prospective imaging clinical trial and 1 (3.8%) patient received a MRI of the tumor bed in addition to a regional CT due to positive margins on surgery. An additional patient (n=1, 3.8%) only had MRI of the tumor bed following an extensive negative lymph node neck dissection. MRI of the nodal basin was utilized for 2 (6.5%) patients, 1 with hidradenitis suppurativa and 1 recurrent tumor with PNI, and PET/CT was utilized in 2 (6.5%) patients, 1 with questionable margin status and 1 with calvarial bone invasion.

The most common imaging schedule was every 6 months for 2 years (n=19, 61.3%). Other schedules included every 4 months for 2-3 years (n=5, 16.1%), every 6 months for 3 years (n=3, 9.7%), every 3-4 months for the first year and every 6 months for years 2-3 (n=3, 9.7%), and every 3 months for 3 years (n=1, 3.2%). More high risk or recurrent tumors received intensified imaging surveillance protocols.

### Regionally metastatic

Of the 34 patients with RM disease, 16 (47%) had IPM, 14 (41.2%) had NM, and 4 (11.8%) had ITM. 3 (8.8%) underwent surgery monotherapy and 31 (91.2%) underwent surgery in addition to neoadjuvant or adjuvant therapy: 8 (25.8%) with neoadjuvant immunotherapy, 7 (22.6%) with neoadjuvant cemiplimab and ART, 6 (19.4%) with chemosensitizers and ART, 5 (16.1%) with ART, and 5 (16.1%) with neoadjuvant and adjuvant cemiplimab.

CT of the nodal basin was the most frequent surveillance imaging modality (n=22, 64.7%) and 12 (35.3%) CLL patients had PET/CT only. Of the 22 patients who underwent a CT scan, 4 (18.2%) solid organ transplant recipients who also had a PET/CT. Most patients underwent surveillance imaging every 6 months for 3 years (n=23, 67.6%). Other imaging schedules included every 3 months for year 1 and then every 6 months for years 2 and 3 (n=9, 26.5%), which was more common for patients with ITM and NM (n=6, 66.7%) or patients with RM in addition to a more aggressive primary tumor (n=3, 33.3%). A less common imaging schedule was every 6 months for year 1 and every 12 months for years 2-3 (n=2, 5.9%) for patients with no additional risk factors.

### Unresectable Disease

Of the 19 (22.6%) unresectable CSCCs, 7 (36.8%) had locally advanced (LA) disease and 12 (63.2%) had metastatic disease. Of the 7 patients with LA CSCC, 6 (85%) were treated with palliative immunotherapy and 1 (14.3%) with cetuximab. The 12 patients with unresectable metastatic disease were treated with systemic therapy either without radiation therapy (n=10, 83.3%) or with radiation therapy (n=2, 16.7%).

Following cessation of treatment, the most common surveillance imaging was CT of the nodal basin (n=11, 57.9%) and PET/CT (n=8, 42.1%) regardless of treatment. Of the 11 that received CT, 6 (54.5%) patients also received PET/CT. The most common surveillance imaging protocols were every 3 months for year 1 and then every 6 months for years 2 and 3 (n=10, 52.6%), every 1-4 months (n=5, 26.3%) for 3 years for patients at highest risk for disease recurrence, and every 6 months for 3 years (n=4, 21.1%) for lower risk patients.

### Proposed Imaging Surveillance Protocols

**Figure 1:**
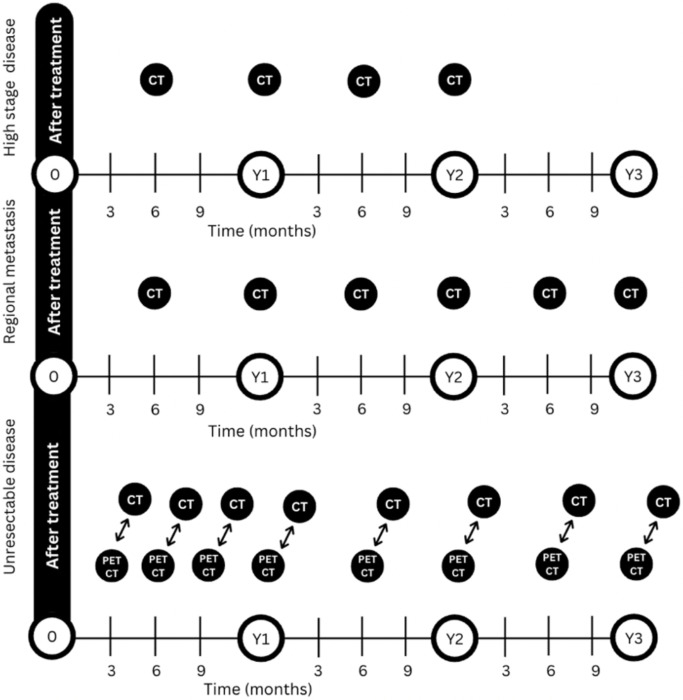
Recommendations for surveillance imaging modalities and timeframes for patients with HS/RM/UR-SCC after curative treatment. *CT*, Computed Tomography, *PET/CT*, Positron Emission Tomography/Computed Tomography.

Based on the clinical experience, surveillance imaging recommendations were developed (Figure 1). For HS disease, we recommend CT every 6 months for 2 years. For RM, we recommend CT every 6 months for 3 years. For UR disease, we recommend PET/CT or CT every 3 months for the first year and then every 6 months for years 2-3.

### Follow up

Out of 84 patients, 53 (63.1%) had at least two years of follow-up. While we are not fully able to assess the utility of imaging surveillance given the incomplete follow up, 4 of the 53 (7.5%) patients had CSCC recurrence (local recurrence: 1 (25%); NM: 3 (75%)) detected radiologically during surveillance. Of these, 3 patients (75%) and 1 (25%) had imaging performed with CT and MRI, respectively. Recurrence was detected by radiologic imaging from 1 to 28 (median: 5) months after primary treatment. One (1.9%) additional patient developed a local recurrence that was detected clinically.

### Deviations from proposed protocols

Several deviations from the proposed surveillance guidelines were observed, with modifications in both imaging frequency and modality (Table III). MRI was obtained in lieu of CT for patients with positive surgical margins, after extensive disease was removed surgically, for monitoring radiographic perineural spread, and for CSCC arising in the setting of hidradenitis suppurativa to help distinguish active tumor from chronic inflammation. PET/CT was performed in lieu of CT when there was high concern for metastasis including suspected distant metastasis or progressive disease, in patients with CLL for whom distinguishing CLL lymphadenopathy from CSCC can be challenging, and in select solid organ transplant recipients with concern for distant metastasis.

## Discussion

Imaging surveillance in treated HS, RM, and UR CSCCs varied by disease extent and treatment. While the most common imaging modality was CT scans every 6 months for 2-3 years, other modalities were utilized at varying frequencies depending on the tumor and patient characteristics.

Multiple imaging modalities have been evaluated for their utility in CSCC. In a systematic review of 34 studies, CT demonstrated high accuracy for detecting bony invasion, with pooled sensitivity and specificity of 75.7% and 98.6%, respectively, and demonstrated the highest sensitivity (96.4%) and specificity (100%) for identifying NM when compared with PET/CT and US^6^. In the same review, MRI showed 94.9% sensitivity and 99.7% PPV for detecting perineural spread. In a study of 233 patients with 246 high-risk CSCCs, US identified subclinical NM with 82% sensitivity (95% CI, 48–98) and 79% specificity (95% CI, 73–84), outperforming clinical exam^7^. Evidence supports the role of PET/CT in recurrent CSCC. In 100 patients undergoing 115 PET/CT scans, 84% showed recurrence, and over one-third revealed previously unrecognized disease, including locoregional or distant metastases, cutaneous lesions, and even second primary malignancies^8^. PET/CT also proved valuable in immunocompromised patients; in a case series of 5 patients with CSCC and CLL, it achieved 99.6% specificity (95% CI, 98–100) in distinguishing CSCC from leukemic infiltration^9^. The heterogeneity of published literature underscores the importance of structured, risk-adapted imaging strategies in the radiologic surveillance of CSCC.

Prior studies have shown that the majority of CSCC outcomes occur within the first three years following diagnosis and treatment, which supports the findings of the present study. A multicenter multinational cohort study of 740 patients with 782 primary CSCCs found that 60.0% of local recurrences, 67.6% of nodal metastases, and 62.8% of satellitosis or in-transit metastases occurred within the first year^11^. By year three, the cumulative incidence rose to 88.9%, 94.2%, and 93.6%, respectively. However, it is important to note that these findings are based on data collected prior to the widespread use of immunotherapy. As immunotherapy continues to reshape the treatment landscape for advanced CSCC, the timing and pattern of recurrences may shift, warranting ongoing reassessment of optimal surveillance intervals.

This study is subject to several limitations. As a retrospective analysis from a single high volume cancer center, findings may not be generalizable to all clinical settings. Additionally, differences in imaging utilization are influenced by provider preferences, institutional protocols, patient medical insurance, and patient factors such as comorbidities and treatment tolerability. Given the size of the cohort, we were unable to compare the efficacy of the modalities.

In summary, we described imaging surveillance in HS, RM, and UR CSCC and propose protocols based on frequently used strategies in our population. These protocols reflect real-world practices tailored to disease severity and treatment type, particularly within the first three years post-treatment, when the majority of recurrences and metastases occur.

## Figures and Tables

**Table I.** Patient and primary tumor characteristics, and staging

Category	Count
Patient characteristics	Total (n=84)
Age, mean (SD), years	74.4 (10.1)
Sex, male	68 (81%)
Immunosuppression	25 (29.8%)
Reason for immunosuppression	
Hematologic malignancy	15 (60%)
Solid organ transplant	8 (32%)
Rheumatoid arthritis	1 (4%)
Chronic prednisone use	1 (4%)
Disease Level at Presentation	
High stage	31 (36.9%)
Regional metastasis	34 (40.5%)
Parotid metastasis	16 (47%)
Nodal metastasis	14 (41.2%)
In-transit metastasis	4 (11.8%)
Unresectable disease	19 (22.6%)
Metastatic disease	12 (63.2%)
Locally advanced	7 (36.8%)
High Stage tumor characteristics	
Location on head or neck	24 (77.4%)
Primary tumor diameter	
<2cm	7 (22.6%)
>/=2cm	23 (74.2%)
Perineural invasion	18 (58%)
Large caliber	11 (61.1%)
Lymphovascular invasion	1 (3.2%)
Differentiation	
Well	9 (29%)
Moderate	6 (19.4%)
Poor	15 (48.4%)
Invasion beyond subcutaneous fat	13 (41.9%)
BWH staging	
T2a	5 (16.1%)
T2b	22 (71%)
T3	4 (12.9%)
AJCC 8 staging, 8^th^ edition	
T1	2 (6.5%)
T2	8 (25.8%)
T3	20 (64.5%)
T4a	1 (3.2%)

*AJCC,* American Joint Committee on Cancer, *BWH,* Brigham and Women’s Hospital, *SD,* Standard Deviation.

**Table II.** Surveillance imaging modalities by disease level

Characteristics	n (%)
High stage disease	31 (36.9%)
CT	26 (83.9%)
MRI	3 (9.7%)
PET/CT	2 (6.5%)
Regional metastasis	34 (40.5%)
CT	22 (64.7%)
PET/CT	12 (35.3%)
LA/RM Unresectable	19 (22.6%)
CT	11 (57.9%)
PET/CT	8 (42.1%)

*CT,* Computed Tomography, *LA,* Locally Advanced, *MRI,* Magnetic Resonance Imaging, *PET/CT,* Positron Emission Tomography/Computed Tomography, *RM,* Regional Metastasis, *US,* Ultrasound.

**Table III.** Clinical scenarios to consider deviation from the proposed imaging surveillance protocol

MRI performed instead of CT	PET/CT performed instead of CT
Positive margins following surgery	High concern for metastasis
Extensive disease removed surgically	Chronic lymphocytic leukemia
CSCC arising in hidradenitis suppurativa	Select solid organ transplant recipients with concern for distant metastasis
Monitoring of perineural spread	Suspected distant metastasis or progressive disease

*CLL*, chronic lymphocytic leukemia, *CSCC*, cutaneous squamous cell carcinoma, *CT*, computed tomography, *MRI*, magnetic resonance imaging, *PET/CT*, positron emission tomography/computed tomography.
